# Investigating site-selection mechanisms of retroviral integration in supercoiled DNA braids

**DOI:** 10.1098/rsif.2021.0229

**Published:** 2021-08-25

**Authors:** G. Forte, D. Michieletto, D. Marenduzzo, E. Orlandini

**Affiliations:** ^1^ SUPA, School of Physics and Astronomy, Peter Guthrie Tait Road, University of Edinburgh, Edinburgh EH9 3FD, UK; ^2^ MRC Human Genetics Unit, MRC Institute of Genetics and Cancer, University of Edinburgh, Western General Hospital, Edinburgh EH4 2XU, UK; ^3^ Dipartimento di Fisica e Astronomia and Sezione INFN, Universitá degli Studi di Padova, 35131 Padova, Italy

**Keywords:** DNA modelling, topology, supercoiling, DNA integration, DNA braids

## Abstract

We theoretically study the integration of short viral DNA in a DNA braid made up by two entwined double-stranded DNA molecules. We show that the statistics of single integration events substantially differ in the straight and buckled, or plectonemic, phase of the braid and are more likely in the latter. We further discover that integration is most likely close to plectoneme tips, where the larger bending energy helps overcome the associated energy barrier and that successive integration events are spatio-temporally correlated, suggesting a potential mechanistic explanation of clustered integration sites in host genomes. The braid geometry we consider provides a novel experimental set-up to quantify integration in a supercoiled substrate *in vitro*, and to better understand the role of double-stranded DNA topology during this process.

## 1. Introduction

Retroviruses, such as HIV, are common pathogens with a significant medical and economic impact on society [1]. In spite of decades of research, there is still a lot that is poorly understood regarding the mechanisms of retroviral infection [2]. One of the essential steps in the infection process is the integration of the viral DNA (vDNA) into the host genome. The integration step is mediated by the intasome, a nucleoprotein complex containing the viral enzyme integrase and vDNA ends, each one carrying a copy of the long terminal repeat [3,4]. Once both 3’-dinucleotides have been removed from the vDNA ends, the intasome together with the tDNA forms a target capture complex (TCC) whose integrase catalyses the strand transfer between the viral DNA ends and the tDNA, resulting in the strand transfer complex (STC). An outstanding challenge in this field is understanding how the three-dimensional organization of the tDNA *in vivo* affects these integration steps [5,6]. Importantly, it is by now well established that different families of retroviruses display different preferences for integration sites [7] and that the selection of the latter is non-random. Mechanistic understanding of integration site selection is important, because it can potentially inform more efficient cures against retroviruses, as well as new strategies for gene therapy (which often employs retroviruses as carriers to deliver desired genes into the nucleus).

In eukaryotes, DNA and histone proteins associate thereby assembling the chromatin fibre which, at short length scales (approx. 10−30 kb or 10–30 nm), is thought to assume heteromorphic conformations, such as one- or two-start helices [8–11]. At larger length scales (approx. 100 kb–1 Mbp or 100 nm) chromosome conformation capture (HiC) revealed the presence of ‘topologically associated domains’ [12–15]. At even larger scales (1 μm), chromosome territories emerge [16]. This hierarchical and non-random organization is thought to affect the site selection of integration events [17–19], yet quantitative assays dissecting the role of each of these levels of organization on the integration selection are largely lacking to date. Most of the existing *in vitro* biophysical assays investigate the effect of naked DNA features, such as flexibility [20] and intrinsic curvature [21].

Recent works have allowed the investigation of the relationship between tDNA organization and retrovirus integration. Prototype foamy viruses (PFVs) use one-dimensional (1D) diffusion mechanisms to enhance the search for integration sites [22] and it was found a greater (two- to fivefold) integration efficiency into supercoiled substrates with respect to torsionally relaxed ones [22,23]. Moreover, magnetic tweezers (MT) assays have been used to study the strand transfer time [22] and the stability of TCCs and STCs [23]. Interestingly, STCs were found to be stable even when large pulling forces were applied to the tDNA tethered between the two magnetic beads composing the MT set-up. This stability is also facilitated by the presence of auxiliary binding interfaces [23,24] allowing the viral intasome to bind the supercoiled tDNA while relaxing torsional stress. The preference for retroviruses to integrate into supercoiled tDNA is in line with the known fact that DNA substrates displaying pre-bent and distorted regions are preferentially targeted by lentiviruses [7,25], including HIV [20,21], and foamy viruses, such as PFV [7].

**Figure 1.**
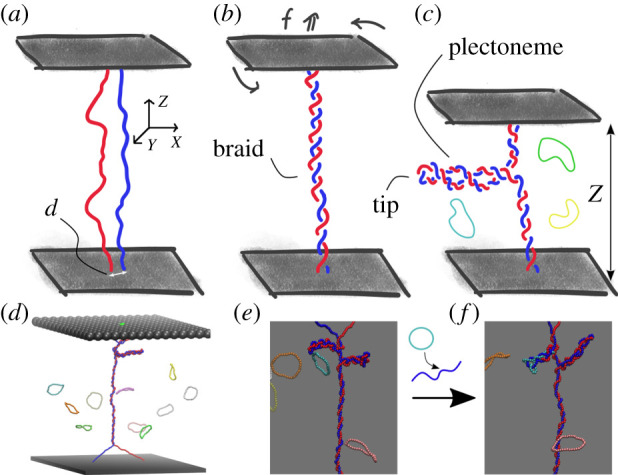
Retroviral integration in DNA braids. (*a*) Two chains representing two dsDNA molecules (one coloured red and the other blue to ease visualization) are tethered to two impenetrable walls. (*b*) The top wall is rotated *n* times to introduce a catenation between the dsDNA strands equal to Ca = *n*, while a force *f* is simultaneously applied to stretch the braid. (*c*) For Ca larger than a critical Ca*(*f*), the braid buckles and forms plectonemes (see electronic supplementary material, figure S1, for the corresponding phase diagram). (*d*) A snapshot from BD simulations of the system under investigation. (*e*,*f*) Example of an integration event, illustrated by successive snapshots in a BD simulation.

In spite of this, the precise location at which retroviruses integrate their genetic material as a function of mechanical stress is still debated. Here, we propose and theoretically analyse a different single-molecule biophysical assay. We consider two (torsionally unconstrained) dsDNA molecules whose ends are anchored to walls (or equivalently to macroscopic beads of a MT) and interwoven to form braids of fixed linking number, or catenation Ca (figure 1). This system is known to display a buckling transition at a critical catenation number Ca* which depends on the stretching force *f*, i.e. Ca* = Ca*(*f*) [26–30]. For Ca < Ca*(*f*), the braid is straight whereas for Ca > Ca*(*f*) the braid buckles and forms plectonemes that resemble those shown by single dsDNA molecules in MT assays [31–33].

The strength of this simple approach is that, on top of simulating the dynamics of plectonemes in braided DNAs [30], we can monitor the precise integration site and the integration efficiency as a function of the supercoiling injected in the braid. Even if this set-up shows some differences with respect to a single supercoiled ds-DNA, its investigation should shed light on properties such as the preferred position of the integration sites, the statistics of repeated integration events and the interplay between supercoiling and integration that are hardly accessible in current experiments. As a result, our simulations can be used to build a mechanistic model for retroviral integration in supercoiled substrates. Additionally, single-molecule experiments studying integration in a DNA braid, which we study here, are in principle possible, and we hope they can be performed in the future to test our predictions.

## 2. Material and methods

Integration of vDNA into DNA braids is simulated via Brownian dynamics (BD) simulations and a sketch of the set-up is shown in figure 1. Two dsDNA molecules with 250 beads each (each bead of size *σ* represents 2.5 nm or 7.3 bp) are tethered to two walls, with the top wall being spun in order to interweave the molecules to form a braid characterized by a desired catenation number Ca, which is a key parameter in our model. The catenation number is kept constant throughout a simulation and a stretching force *f* is applied to the top wall (figure 1). A Kratky–Porod potential is used to impose a persistence length equal to *l*_*p*_ = 20*σ* = 50 nm, which is typical for dsDNA [34]. Viral DNA is modelled as circular chains composed of 40 beads, or about 300 bp. This length is chosen so that it is larger than the persistence length of DNA but short enough to allow efficient and feasible simulations. In each simulation, we introduce 10 vDNA rings which can integrate into either of the two dsDNA molecules. The numerical density of rings in the simulation box ranges between 10^−4^*σ*^−3^ and 10^−3^*σ*^−3^: these low concentrations are chosen in order to minimize the probability of simultaneous integration events, and to be able to study correlations between consecutive integration events. The stochastic integration process is simulated by using a Monte Carlo algorithm: whenever a pair of bonds, one belonging to the tDNA braid and the other to the vDNA, are proximate in three dimensions, a swap between them is attempted in a single simulation step [19,35]. The attempted swap is then accepted or rejected based on the difference in energy between the initial and final conformations, i.e. according to a Metropolis scheme with probability *p* = exp(−(*E*^′^−*E*)/*k*_*B*_*T*), where *k*_*B*_ is the Boltzmann constant, *T* the room temperature and *E* and *E*^′^ are the energy of the configurations respectively before and after the bond-swapping move. Additional details are given in the electronic supplementary material.

## 3. Results

### 3.1. Integration affects plectonemic dynamics in the buckled phase

**Figure 2.**
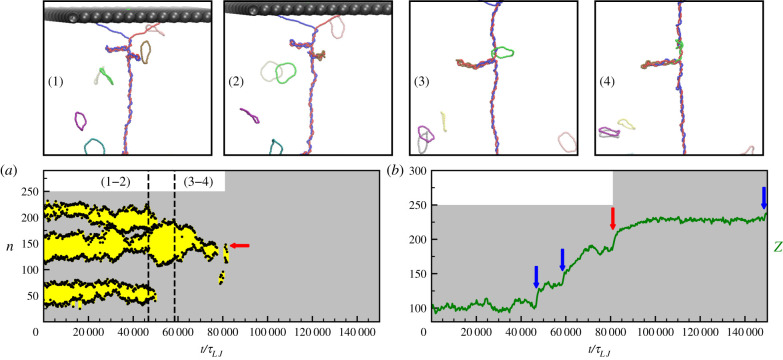
Effect of integrations on braid dynamics in the buckled state. (*a*) Kymograph showing the evolution of plectonemes (monitored on the red DNA, see electronic supplementary material) for a simulation with $f=6\frac{\sigma }{k_{B}T}$ and Ca = 36. Yellow regions represent the segments inside plectonemes while black dots their boundaries. During this run four integration events are observed, three in the blue DNA and one in the red one. The red arrow indicates the moment and the position of the integration in the red strand. The numbers (1), (2), (3) and (4) refer to the top snapshots. Between (1) and (2) there is an integration inside a plectoneme, while between (3) and (4) another integration takes place in the braided part of the system. The grey background shows the step-wise increase in length of the red strand, which is initially equal to 250*σ* (see *y* axis) and it increases at time *t* ∼ 80000 *τ*_*LJ*_ when a ring integrates. (*b*) End-to-end *Z* distance versus time. Blue and red arrows show the moments of integration in the blue and red strand respectively. At each integration event, we observe a jump in *Z*.

For catenation numbers greater than the critical value Ca*(*f*), the system undergoes a buckling transition which lowers the total free energy by converting twist into writhe [27,29,36]. From now on, all simulations will be performed with a pulling force $f=6\;\frac{k_{B}T}{\sigma }$ whose critical catenation is Ca* = 29. This parameter choice is representative of the general behaviour. A buckled state is characterized by the formation of plectonomes which can be identified by computing the instantaneous map of contacts between DNA segments in the same molecule (see figure S1 and electronic supplementary material for details about the algorithm). Plectoneme dynamics are rich, and encompass fusion, fission and 1D Ostwald ripening [30]. In this section, we study how viral integration qualitatively affects the dynamics of plectonemes and braided DNA in the buckled phase. More specifically, we analyse the plectoneme positions (figure 2*a*) and the end-to-end distance *Z* (figure 2*b*) over time, following multiple integration events. When the first integration occurs the length of the blue dsDNA molecule displays a sudden increase, in turn yielding a jump in the end-to-end distance *Z* (figure 2*b*). After each integration, the braid displays a slow relaxation to a new steady state in *Z*, which can be resolved if integration events are sufficiently spaced in time. Concomitantly with the increase in end-to-end distance, plectonemes decrease in number and size following integration. Thus, the simulation shown in figure 2 started with three stable plectonemes, but after three integrations none remained. The dynamical pathway through which plectonemes disappear is nontrivial. For instance, immediately after the first integration (between snapshots (1) and (2) in figure 2) a fusion and a hopping event result in the formation of a single larger plectoneme whose size is reduced when the second integration occurs. The reason why plectonemes disappear following successful integration events is that the total length of the braid *L* increases after each event, which reduces both the catenation density Ca/*L* and the ratio of *d*/*L* where *d* is the lateral spacing between the tethering points on the same wall. As shown in previous works [27,29], both these parameter changes drive the system away from the buckled phase.

The qualitative effects of integration on plectoneme and braid dynamics shown in figure 2 could be studied experimentally by using methods similar to those in [37], and we hope that similar studies will be performed in the future. We now proceed to perform a more quantitative investigation of the statistics of single and multiple integration events in our simulations.

### 3.2. Statistics of single integration events

In [22], the authors find that for PFV intasome on stretched single-molecule DNA, more than 97% of the search events do not generate successful integrations. This is in line with the fact that integration events need to significantly distort the DNA substrate [19,21] and hence must overcome a substantial energy barrier.

**Figure 3.**
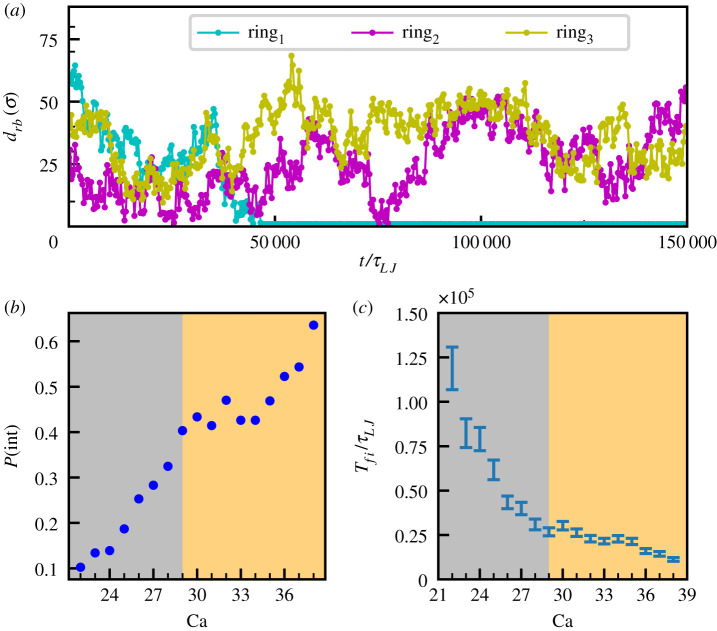
Statistics of single integrations. (*a*) Trajectory of three rings diffusing in the system corresponding to the kymograph in figure 2. The distance between the ring and the braid *d*_*rb*_ is monitored over time: ring_1_ integrates, ring_2_ gets close to the braid three times without integrating, while ring_3_ never gets close to the braid. (*b*) Plot of the integration probability against the catenation number. The grey background refers to Ca < Ca* (straight braid phase), while the orange one to Ca ≥ Ca* (buckled phase). It is interesting to note that the curve significantly changes its slope in proximity of Ca*. (*c*) Plot of the average integration time (i.e. the average time to the first successful integration) as a function of Ca. The higher the catenation number, the shorter the time needed to observe the first integration.

With our model, we can explore this process in more detail and on braided structures. In figure 3*a*, we report the trajectories of three vDNA rings for the simulation represented in figure 2 described in terms of their minimal distance *d*_br_ from the braid. It can be noted that, while ring_1_ integrates as soon as $d_{\mathrm{br}}\leq d_{\mathrm{br}}^{*}=1.5\sigma$ (the maximum distance at which integration may occur), ring_3_ never gets sufficiently close to the braid to attempt an integration ($d_{\mathrm{br}}\geq d_{\mathrm{br}}^{*}\;\forall t$). Interestingly, ring_2_ displays a sufficient small distance *d*_br_ multiple times, but it never integrates. This highlights the stochastic nature of integration, which we now address more quantitatively by first analysing the statistics of single integration events. In particular, we are interested in understanding how the searching and integration efficiency of the vDNA rings depend on the braid configuration, and on whether the braid is in the straight or buckled phase. To quantify these features, we perform 150 independent simulations in which 10 vDNA rings can diffuse within the system and integrate into substrates with 22 ≤ Ca ≤ 38. We count the number of times any of these rings reaches a distance $d_{\mathrm{br}}\leq d_{\mathrm{br}}^{*}$ and identify this quantity as the number of integration attempts *N*_attem_ (in line with [22]). Denoting by *N*_int_ the number of successful integration events we compute the integration probability as the ratio *P*(int) = *N*_int_/*N*_attem_ averaged over all trajectories. This is reported as a function of the catenation number Ca in figure 3*b*. The overall trend is that the integration probability increases with Ca. Note that at low catenation number just 10% of the vDNA rings approaching the braid actually integrate, while for higher Ca we observe up to 60% of successful integrations. This is in line with the experiments performed in [22] where authors observed that 90–97% of the times PFV intasome search events on stretched non-supercoiled DNA fail to produce successful integrations and that supercoiling generally increases this integration probability between two- and fivefold. The agreement is significant, given that we are considering a different system (braided DNA instead of single supercoiled double-stranded DNA) and that we are using a simple coarse-grained model for both tDNAs and vDNA rings. Intriguingly, we also find that the slope of *P*(int) versus Ca flattens in proximity of the critical value separating the straight and buckled phase (Ca* = 29), which may underlie a qualitative difference between the integration in the two phases.

The catenation number Ca also strongly affects the time needed for integrations to take place. In figure 3*c,* we show that the average time to integrate into the braid for the first time, *T*_*fi*_, is shorter for larger catenation numbers, again highlighting the fact that plectonemic supercoiling aids integration, making it kinetically faster as well as more likely. An explanation of the trends observed in figure 3*b*,*c* will be given in the next paragraphs.

### 3.3. Statistics of multiple integration events

Within our simulations, we can explore multiple rounds of integration events within the same braid, and therefore investigate cooperative effects and spatial correlations between subsequent integration events. That such correlations may exist in reality is suggested by the observations of clustered distributions of retroviral integration sites [38] and transposable elements [6] in the genome.

To quantify how an integration event affects the next one, we performed simulations for braids with catenation number 22 ≤ Ca ≤ 38 where we only allowed two rings to integrate. The results of these simulations show that for each Ca the probability that the two integrations occur in the same molecule is $p_{ss}≃70\text{\%}$. Instead, in the case of random events, the first integration would yield a 40 *σ* increase in the strand length and the second ring would just have a ∼54% probability of integrating into the same strand.

**Figure 4.**
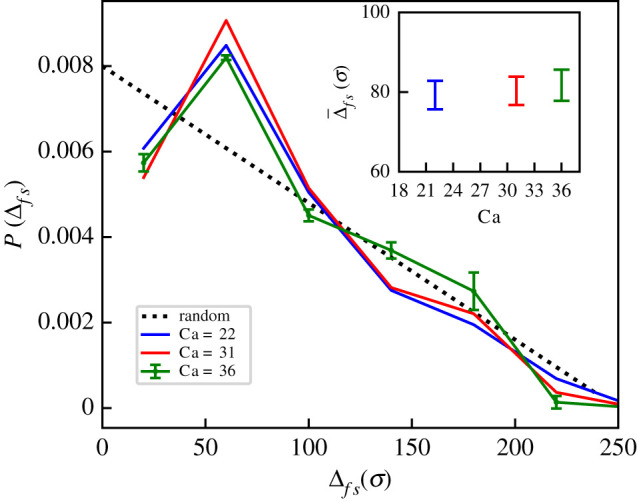
Statistics of multiple integrations. Probability distribution for Δ_*fs*_—the distance along the braid between sites of successive integrations—for three different values of Ca compared to the case in which integrations occur randomly along the polymer, i.e. with a probability *P*_0_(*x*) = (2(*L*−*x*))/*L*^2^. For the case Ca = 36, we report also the errorbars. We observe a maximum for intermediate values of Δ_*fs*_, suggesting that there is a favoured typical spatial separation between successive integration events. As a convention, if the integration breaks the bond between the beads with ID number *id*_1_ < *id*_2_, we identify the integration site with *id*_1_. Inset: average of the contour distance Δ_*fs*_ between successive integrations for the same three values of Ca used in the main figure.

By analysing the distance along the braid backbone separating the sites of two subsequent integration events, which we call Δ_*fs*_, we find that the corresponding distributions are significantly different from those expected for random, uncorrelated, integration events, for which we expect a distance distribution given by *P*_0_(*x*) = (2(*L* − *x*))/*L*^2^ (figure 4). The curves in figure 4 do not depend appreciably on the exact values of Ca (data correspond to Ca = 22, 31, 36), and all show a maximum for intermediate distances, i.e. for Δ_*fs*_ ∼ 50*σ*. We interpret this effect as due to the transient creation of a writhing segment in proximity of the first integration; this effectively favours a second integration in close proximity to the first before the excess in contour length relaxes.

### 3.4. The spatio-temporal pattern of bending energy explains the integration statistics

Integration events in our model are associated with an energy barrier, which is related at least in part to the need to locally bend the DNA molecule where the retrovirus gets incorporated [19] (see electronic supplementary material, figure S3). We therefore ask to what extent we can understand the nontrivial integration statistics described in the two previous sections by analysing the spatio-temporal pattern of bending within the braid, as this should determine the energy barrier along the latter. To answer this question, we first monitor the total bending energy (per bead) of each braided DNA molecule, which can be computed as

**Figure 5.**
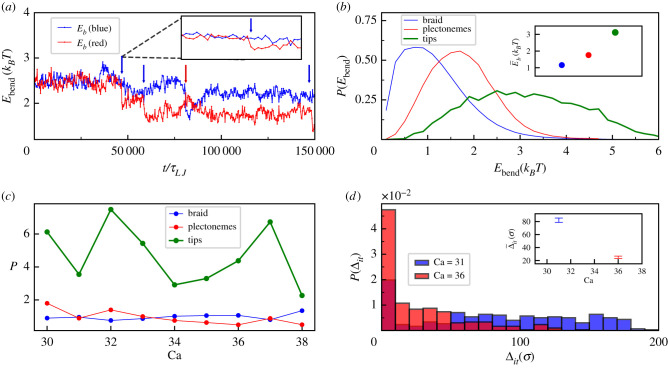
Bending energy and integration sites. (*a*) Bending energies per bead for the same run reported in figure 2. Blue and red curves are bending energies *E*_blue_, *E*_red_ of the blue and red DNA, respectively. Blue and red arrows indicate the moments of integration events. (*b*) Histograms of local bending energies per bead in the braided part (blue), plectonemic part (red) and plectonemic tips (green). (*c*) Integration probabilities in the braided part (blue), plectenemic part (red) and plectonemic tips (green), normalized by the number of beads involved in each of the three motifs, versus Ca. (*d*) Distribution of Δ_*it*_, the distance between the integration site and the tip of the closest plectoneme, for two values of the catenation number Ca = 31 and Ca = 36. In both cases there is a peak close to zero, indicating that rings prefer to integrate near plectonemic tips. Inset in (*a*) represents a zoom, while insets in (*b*,*d*) report the average values of the quantity plotted in the corresponding panel.

3.1 $$E_{b}(x)=\frac{k_{B}Tl_{p}}{L\sigma }\sum_{i=2}^{L-1}[1+\cos\theta_{i}]\;\mathrm{with}\;x=\{\mathrm{red},\mathrm{blue}\},$$

where *θ*_*i*_ is the angle between two consecutive pairs of beads having bead *i* in common. In figure 5*a,* we report the value of *E*_*b*_(*x*) for the trajectory shown in figure 2. Note that, in response to the first integration occurring on one molecule (labelled ‘blue’) the bending energy of the second molecule, *E*_*b*_(red), drops markedly, indicating that the second dsDNA molecule (labelled ‘red’) becomes straighter. Similarly, an integration on the red strand produces a sudden drop of the bending energy of the blue counterpart, *E*_*b*_(blue). These findings indicate that integrations within one dsDNA render the molecule effectively floppier and hence easier to deform further. This explains the observed persistence of multiple integrations on the same molecule: the first integration breaks the symmetry between the two DNAs thereby reducing the effective persistence length of the one where integration took place (figure 5*a*). Thus, it is natural to expect that a second integration will preferably occur in the same, now more flexible, DNA where the elastic energy barrier to overcome is smaller [19]. It is worth noting that this result holds for a system composed by a dsDNA braid and it cannot be compared directly to MT experiments performed on a single dsDNA molecule. In the latter case, indeed, a retroviral integration cuts both strands of the dsDNA molecule leading to supercoiling relaxation. Consequently, the flexibility of the molecule decreases, as the latter is tethered between the two magnetic beads [23]. Since variations in the bending energy favour multiple integrations within the same strand, it is of interest to ask whether they can also affect the specific location (along the backbone) where integrations occur. To see whether this is the case, we compute the average bending energies for the different geometrical motifs present in the system: the *braids*, the *plectonemes* and their *tips*. Note that in the straight braid phase only the braided part is present, while in the buckled phase a typical configuration can be described as an alternating sequence of braids, plectonemes and tips (figure 1). In figure 5*b,* we show the distributions of the bending energy for the three motifs: as expected, the straight braids have the smallest bending energy, plectonemes carry intermediate values whereas the largest bending energy is stored at the tips. In light of what seen above, it is natural to expect that integrations may occur more likely in regions with higher bending energies (i.e. more prone to thermal deformations) and so in plectonemes and tips. To verify this prediction, we classify all integration events according to the region in the braid where they occur, and report the results in figure 5*c*: the data show that the integration probability within tips is the largest, while integrations into braids or plectonemes have a similar probability. It is important to note that this analysis identifies a tip as the five beads surrounding the centre of mass of a plectoneme. This is just an arbitrary choice as there is not an objective way to define tips. Nonetheless, it might happen that in different set-ups (i.e. different Ca) the tip size changes producing the presence of peaks in the green curve of figure 5*c*.

The preference for tip integration can alternatively be quantified by analysing the distribution of the distance between the integration sites and the nearest plectoneme tip, which we call Δ_*it*_ (figure 5*d*). The data show that the larger the value of the catenation number, the stronger the preference tends to be. We can further rationalize the statistical preference for integration at plectoneme tips by computing the local curvature of a tip of length *l* as a function of temperature *T* and external stretching force *f*. This can be done by minimizing the free energy

3.2 $$\frac{F}{k_{B}T}=\frac{2l_{p}\zeta }{l}+\frac{\;fl}{k_{B}T}$$

with respect to *l*. In equation (3.2), the first term is the elastic bending energy required to bend the tip into a tear-drop shape (*ζ* = 16) while the second term accounts for the fact that the tip does not feel the effect of the stretching force. Minimization of the free energy gives $l=\sqrt{k_{B}T2\epsilon l_{p}/f}$ and a local curvature $\kappa ∼1/l≃\sqrt{\;f/(k_{B}Tl_{p})}$ [29]. This means that higher forces and smaller persistence lengths (or even mismatches and defects [32,33,39]) increase the curvature of the tip and hence the probability of integrations within this motif.

The preference for integration within the tip might also qualitatively explain the entropic selection mechanism for the separation between successive integration events (figure 4). Indeed, immediately following an integration event, the region of the braid where this has occurred has extra uncompensated contour length in one of the DNAs, which does not feel the tension from the stretching force before the braid reorganizes locally. Because stretching suppresses writhing, the transient absence of stretching will favour local writhing and this writhe excess might, in turn, attract retroviral DNA during the timescale of writhe relaxation, hence causing the observed cooperativity between successive integration events (see electronic supplementary material, movie S1). The role that the local bending energy profile has in the integration process can also account for the statistics of single and multiple integration events reported in figure 3. For instance, the monotonic increase in successful integrations versus attempts, *N*_int_/*N*_attem_, with Ca can be explained by noting that increasing the catenation number leads to a linear increase in the average amount of writhe in the braid, given the decomposition Ca = *Tw* + *Wr* [40]. In turn, as the increased writhe translates into an increase in local DNA bending energy (see electronic supplementary material, figure S3), it also leads to an increase in the integration probability and to a decrease in integration time (as seen in figure 3*b*,*c*).

The model considered here does not include a 1D diffusing sliding mechanism (i.e. along the backbones of the two chains) as experimentally observed in [22]. However, the details of the diffusive process—whether it is three-dimensional diffusion in the bulk, 1D diffusion along the braid, or a combination of the two as in facilitated diffusion—should not change either the integration probability *P*_int_ (figure 3*b*) or the relative probabilities of having integrations in tips, plectonemes and braided parts (figure 5*c*) as long as we use the same simulation parameters. In line with these considerations, we also expect that 1D diffusive sliding mechanism could reduce the integration time *T*_*fi*_ at fixed catenation number, but should leave the general behaviour in figure 3*b*,*c* unaltered.

## 4. Conclusion

To summarize, in this work, we used BD simulations to study the integration of viral DNA in a DNA braid made up by two interwoven double-stranded DNA molecules. With these simulations we could study the relationship between supercoiling and integration efficiency, investigating the statistics of the exact integration site. We have found that integration events in buckled braids qualitatively affect the dynamics of plectonemes, because they modify the length of one or both the polymers in the braid, pushing the system away from the buckled phase dynamically (figure 2). Such qualitative effects should be experimentally testable by microscopy (e.g. by using similar methods as in [37]), or via optical tweezer experiments measuring the braid extension as a function of time.

We have also quantified the statistics of single and multiple integration events in the braid. Our results show that integration is facilitated by supercoiling and writhing, as the integration probability increases in the buckled phase, and the typical integration time concomitantly decreases sharply. Within the buckled phase, integration is also favoured close to the tips of plectonemes. Pleasingly, these results are quantitatively in line with those found experimentally for integration in *single* supercoiled DNA substrates [22]. An analysis of the pattern of local bending energies along the braid for different values of the catenation number shows that these determine in large part the observed pattern of integration sites. This is because integration requires DNA bending, hence larger local bending energies correlate with lower energy barriers opposing integration (and hence with higher integration probabilities). Thus, the buckled phase has a larger stored bending energy with respect to the straight braid phase, and this is why integration in the former is more likely (and faster). Additionally, the largest bending energy in a braid is stored at plectoneme tips and we predict that retroviruses tend to integrate there more often than at another random position in the braid.

Finally, we predict that successive integration events should be correlated. First, integration in one of the two molecules in a braid breaks the symmetry in the system, and favours successive integrations in the same molecule. Second, in the buckled phase, we have found that there is an enhanced probability for the second integration event to occur at a typical distance from the site of first integration. This is because the first integration event promotes the local increase of writhe. We thus argue that it is more energetically favourable for the second vDNA to integrate in the proximity of the first, while the writhe is relaxing and redistributing along the chain (see also electronic supplementary material, movie S1). Our results, that should be testable with single molecule experiments, provide a first indication of how retroviral integration events are affected by the presence of plectonemic structures in the substrate. Moreover, it would be interesting to perform simulations that include more details about the biology of retrovirus integration. For instance, one can investigate the role played by auxiliary binding interfaces [23,24] to better understand the dynamics of STCs.

## Data accessibility

The data generated to produce the figures in this paper and computer codes can be found at https://git.ecdf.ed.ac.uk/dmichiel/integrations_in_braids.
